# 898. Influenza Surveillance in the US Veterans Health Administration (VHA): 2022-2023 Season

**DOI:** 10.1093/ofid/ofad500.943

**Published:** 2023-11-27

**Authors:** Cynthia A Lucero-Obusan, Patricia Schirmer, Connor W Edson, Gina Oda, Mark Holodniy

**Affiliations:** U.S. Department of Veteran Affairs, Public Health National Program Office, Palo Alto, California; Department of Veterans Affairs, Palo Alto, California; Department of Veterans Affairs, Palo Alto, California; Department of Veterans Affairs, Palo Alto, California; Department of Veterans Affairs, Palo Alto, California

## Abstract

**Background:**

Influenza causes seasonal epidemics with substantial morbidity and mortality. Veterans Health Administration’s (VHA) large elderly population is at higher risk for influenza complications, including hospitalization, and death. Herein we summarize VHA’s national annual surveillance data for influenza activity and vaccinations.

**Methods:**

Influenza-like-illness (ILI) as well as influenza-coded outpatient visits and hospitalizations, laboratory testing, and antivirals were obtained from VHA data sources (10/2/2022-4/22/23) and compared to prior years and CDC data. Vaccinations were captured starting 8/1/2022, with rates calculated based on VHA users in each fiscal year. Influenza RT-PCR positive respiratory samples underwent whole genome sequencing (WGS) to analyze clade and antiviral resistance.

**Results:**

Surveillance metrics are presented (Table). Vaccinations were decreased compared to 2021-22, but the used of adjuvanted vaccine was increased. Weekly ILI ranged from 0.4%-1.4% in primary/urgent care settings. Activity was highest for 2022 Week 48, matching national influenza trends (Fig.1). Testing found 23,297 influenza positives of 457,020 tests performed (5.1%). Median length of stay (3 days) for hospitalized patients and deaths (127, 3%) were stable. Among 115 deaths where influenza type was available, 110 had Influenza A and 5 Influenza B. Following two low activity seasons, total influenza positives, outpatient visits, and hospitalizations during 2022-23 tracked closer to seasons prior to the COVID-19 pandemic (Table, Fig.2). 411 samples (in 16 states) were sequenced (410 Flu A: 136 H1, 274 H3; 1 B/Victoria), representing 12 subclades, which aligned with CDC reporting. No drug resistance mutations were found (Fig.3).

**Figure:**
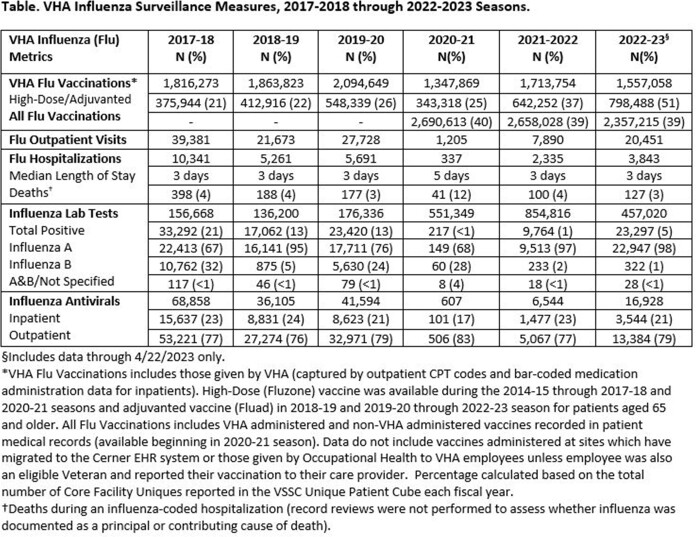

**Figure:**
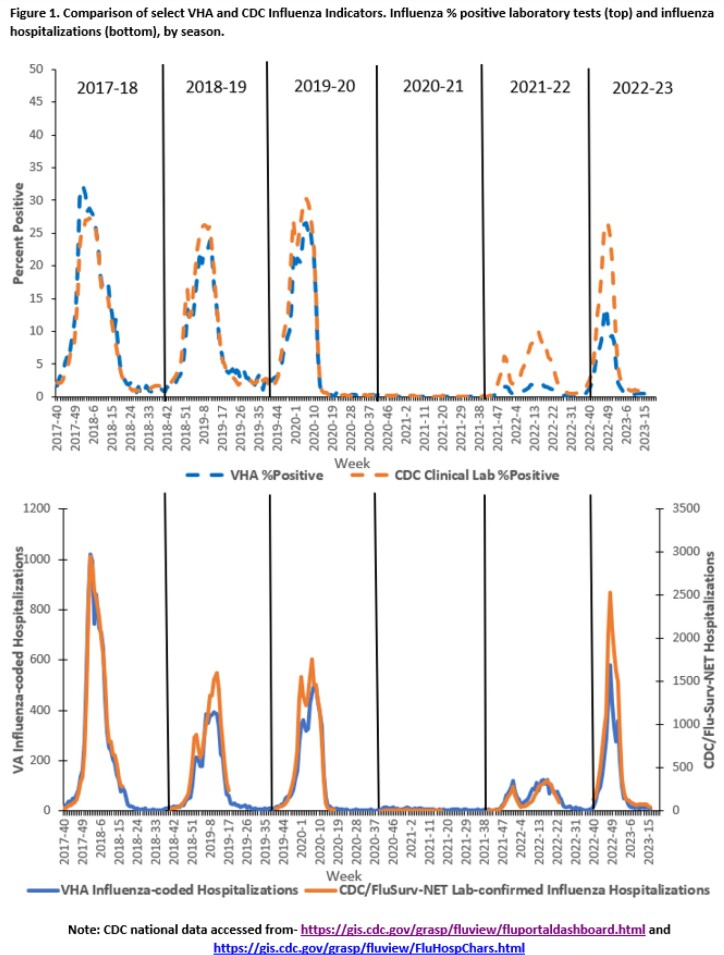

**Figure:**
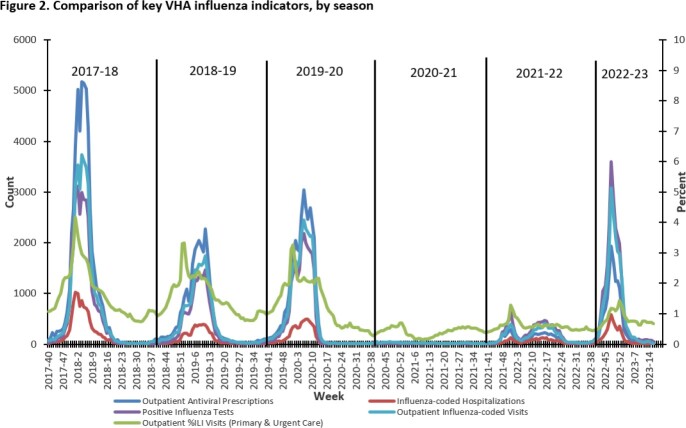

**Conclusion:**

For 2022-23, influenza vaccinations in VHA dropped but percent vaccinated was stable. For the first time, over half of VHA-administered vaccines were adjuvanted formulation. The influenza season was notable for an earlier than usual peak (late Nov/early Dec), with overall increased activity compared to the prior two seasons. Testing for influenza during 2022-23 season was decreased but remained higher than pre-COVID-19 pandemic testing levels. VHA influenza activity and WGS analysis tracks closely with national CDC reporting.

**Figure:**
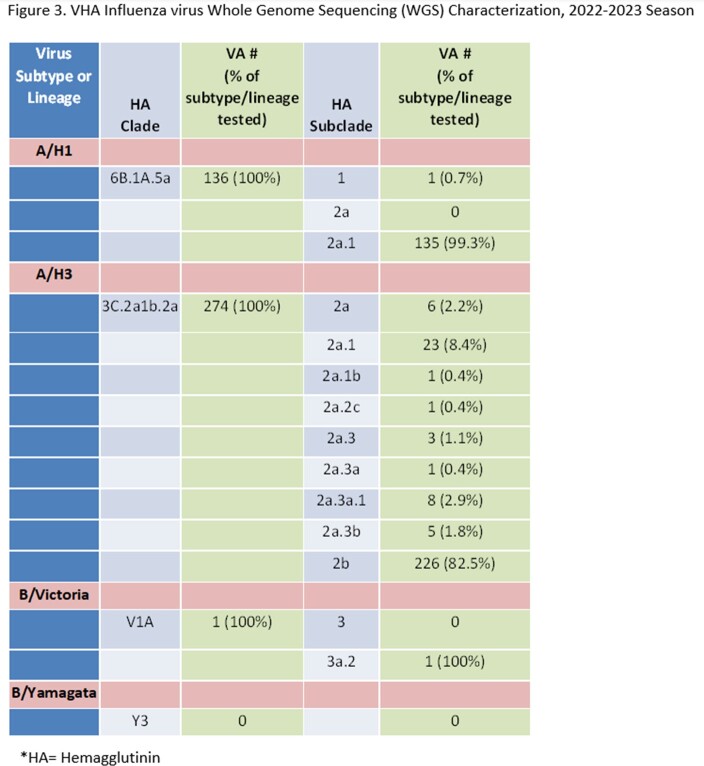

**Disclosures:**

**All Authors**: No reported disclosures

